# Ghana and the COVID-19 pandemic

**DOI:** 10.4314/gmj.v54i4s.1

**Published:** 2020-12

**Authors:** David Ofori-Adjei, Margaret Lartey, Kwadwo A Koram

**Affiliations:** Editorial Office, Ghana Medical Journal editor@ghanamedj.org

A new virus causing predominantly respiratory tract infection was described in China late 2019. The virus was subsequently named the severe acute respiratory syndrome coronavirus 2 (SARS-CoV-2) and the disease it causes as COVID-19. Subsequently the virus spread to many parts of the world. This resulted in the World Health Organisation declaring COVID-19 a global pandemic on 11^th^ March 2020.^1^ The first cases of COVID-19 were reported in Ghana on March 12, 2020. The initial cases of COVID-19 in Ghana were predominantly from international travellers. Subsequently community spread occurred. A national response effort was instituted spearheaded by the Presidency, with emphasis on instituting preventive and curative interventions to limit the spread of the disease. Several interventions including health, regulatory, social, economic and financial strategies were implemented, the key ones being limited lockdown in two metropolitan areas (Accra plus Kasoa and Kumasi), closure of all entry points into the country and limits on number of persons at public gatherings including religious premises and funerals, frequent hand washing with soap under running water, use of alcohol-based sanitizers and social distancing. Guidelines were also developed for treatment^2^ and case management^3^ and mandatory wearing of face masks was later added to the interventions.

National civic and political events – registration for the National Identification Card and a new National Voters' Register and political rallies towards the country's general elections in December 2020 – placed additional strains on the strategies to contain and mitigate the pandemic. In all these events it became imperative that organisers ensured compliance with safety protocols described earlier. The epidemic, however, brought in its wake healthcare delivery challenges including access to healthcare, health workforce challenges, and access to personal protective equipment.

The President insisted in all his updates on the pandemic (21 so far) that decisions on the pandemic will be guided by science. However, specific allocation of funds to institutions to conduct research and generate the local evidence to guide the national response was not obvious and scientists used internal resources to support the response. The limited activities included the modelling of the spread of the epidemic coordinated by the Science Section of the Ghana Academy of Arts and Sciences, the sequencing of the virus circulating in Ghana^4^ by Noguchi Memorial Institute for Medical Research and the West African Centre for Cell Biology of Infectious Pathogens of the University of Ghana and their collaborators.

Identification of new strains of the virus makes it imperative that direct government funding of such research activities be activated. Due to the limited funding of research, other essential research such as the social and economic effects of the response strategies was not conducted, thus limiting the effectiveness of the interventions put in place.

This pandemic offers the health sector opportunities to improve aspects of the healthcare system. Much data has been collected using various data collection systems and these should be consolidated and appropriately linked to form a basis of generating intelligence for decision making, monitoring and evaluation. Individual scientists and scientific institutions have advocated for government support to conduct relevant research to augment the evidence for decision making. It is important that these pleas are adequately addressed. Similar arguments can be made for areas of transmission of the virus, levels of community exposure, health service preparedness, post-COVID-19 complications and psycho-social effects of the pandemic. It is now time for the government to seriously consider the establishment of an institution or network of institutions to support communicable diseases outbreaks research.

The Ghana Health Service took the frontline position in the management of this pandemic in relation to surveillance whereas the teaching hospitals led in case management and the Universities in testing and laboratory surveillance. It became obvious that the Ministry of Health did not appear to have its own stock of technical expertise in various aspects of healthcare. This situation deprives the Ministry of in-house technical expertise to advise and assist the Minister for Health in policy development, its main mandate. This notwithstanding, it may be argued that the situation provides an opportunity to establish a platform that could bring in the required expertise from the agencies of the Ministry and other establishments such as the Universities to work together. It is hoped that such a platform will be ready and available with lean and efficient mechanisms so as to avoid undue delays in developing policies, especially in times of emergencies.

The Ghana Medical Journal put out a call in June 2020 requesting for manuscripts on COVID-19 for consideration in a special issue of the journal. The areas of solicitation included national response, epidemiology, socioeconomic and health costs, clinical care, effect on healthcare system, hypothesis and social media response. In this special issue of the journal, we publish some of the experiences of the Ghanaian response during the first five months of the epidemic. The articles deal with the clinical care, autopsy findings, medical education, epidemiology, socio-economic characteristics and socio-cultural dimensions.

Generally, the response to the pandemic in Ghana has been seen as favourable^5^, however, there is the need to document the processes and principles that drove the response, as well as lessons learnt to inform the management of future disease outbreaks. This issue of the journal seeks to document mainly some of the local experiences in the management of COVID-19 in Ghana. Hopefully, the opportunity exists for a detailed historical account of the pandemic.
